# Left Bundle Branch Area Pacing versus Right Ventricular Pacing in Patients with Atrioventricular Block: An Observational Cohort Study

**DOI:** 10.1155/2023/6659048

**Published:** 2023-08-21

**Authors:** Zhongxiu Chen, Yuanning Xu, Lingyun Jiang, Ran Zhang, Hongsen Zhao, Ran Liu, Lei Zhang, Yajiao Li, Xingbin Liu

**Affiliations:** ^1^Department of Cardiology, West China Hospital of Sichuan University, Chengdu, Sichuan, China; ^2^Information Center of West China Hospital of Sichuan University, Chengdu, Sichuan, China; ^3^Department of Cardiology, Chengdu Xinhua Hospital, Chengdu, Sichuan, China

## Abstract

**Objective:**

We aim to conduct a comparison of the safety and effectiveness performance between left bundle branch area pacing (LBBAP) and right ventricular pacing (RVP) regimens for patients with atrioventricular block (AVB).

**Methods:**

This observational cohort study included patients who underwent pacemaker implantations with LBBAP or RVP for AVB indications from the 1st of January 2018 to the 18th of November 2021 at West China Hospital. The primary composite outcome included all-cause mortality, lead failure, or heart failure hospitalization (HFH). The secondary outcome included periprocedure complication, cardiac death, or recurrent unexplained syncope. A 1 : 1 propensity score–matched cohort was conducted for left ventricular (LV) function analysis.

**Results:**

A total of 903 patients met the inclusion criteria and completed clinical follow-up. After adjusting for the possible confounders, LBBAP was independently associated with a lower risk of the primary outcome (OR 0.48, 95% CI 0.28 to 0.83, *p* = 0.009), including a lower risk of all-cause mortality and HFH. No significant difference in the secondary outcome was detected between the groups except that LBBAP was independently associated with a lower risk of recurrent unexplained syncope. In the propensity-score matching cohort of echocardiographic analysis, the LV systolic dyssynchrony index was lower in LBBAP compared with that in RVP (5.68 ± 1.92 vs. 6.50 ± 2.28%, *p* = 0.012).

**Conclusions:**

Compared to conventional RVP, LBBAP is a feasible novel pacing model associated with a significant reduction in the primary composite outcome. Moreover, LBBAP significantly reduces the risk of recurrent unexplained syncope and improves LV systolic synchrony. This study is registered with ClinicalTrials.gov NCT05722379.

## 1. Introduction

Right ventricular pacing (RVP) is the standard treatment for patients with continuous ventricular pacing needs. However, clinical studies have shown that RVP can cause electrical and mechanical dyssynchrony of the left ventricle and increase the risks of cardiac insufficiency, atrial fibrillation (AF), and death [1–3]. His bundle pacing and biventricular pacing methods emerged as physiological pacing strategies with drawbacks. As His bundle is short, its pacing is difficult, and it requires a right chamber backup electrode because of the high rate of later-period electrode displacement [4]. Biventricular pacing is technically difficult and expensive, and therefore only recommended in chronic heart failure patients with left bundle branch (LBB) block [5]. Left bundle branch area pacing (LBBAP), a novel pacing strategy which paces the LBB area, was first reported as an alternative to left ventricular (LV) resynchronization by Huang et al. in 2017 [6]. Subsequent studies have demonstrated that LBBAP is a safe and feasible pacing method with a low pacing threshold and produces a narrow electrocardiogram (ECG) QRS duration [7, 8]. LBBAP can quickly achieve relatively simultaneous biventricular pacing once captured and is considered to be the most promising physiological pacing method and maybe the optimal pacing mode. However, a direct comparison of the safety, efficacy, and LV systolic synchrony between LBBAP and RVP regimens was rare. In our study, we aim to conduct a comparison of the safety and effectiveness performance between these two pacing methods for patients with atrioventricular block (AVB).

## 2. Method

### 2.1. Study Subjects

**Figure 1:**
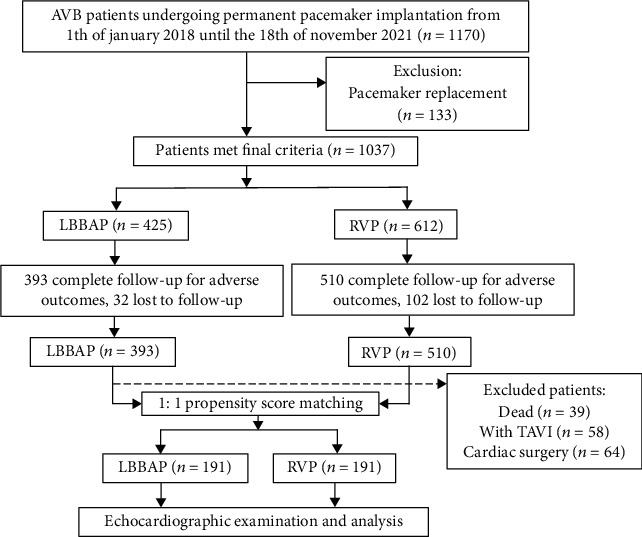
Study flowchart describing inclusion and exclusion criteria leading to the final cohort of patients. AVB: atrioventricular block; LBBAP: left bundle branch area pacing; RVP: right ventricular pacing; TAVI: transcatheter aortic valve implantation.

This single-center observational cohort study included 1170 consecutively high-grade AVB patients undergoing permanent pacemaker implantations from the 1st of January 2018, when the LBBAP was launched, to the 18th of November 2021 at West China Hospital. The study protocol was approved by the Ethics Committee of the West China Hospital of Sichuan University (Sichuan, China). All implantations were performed by senior pacemaker implantation cardiologists at our institution. 133 patients with pacemaker replacements were excluded. The remaining 1037 patients were divided into the LBBAP group and the RVP group according to the procedure of ventricular lead (Figure 1).

### 2.2. Procedure Implantation and Device Programming

LBBAP was conducted according to the previously published procedure [6]. In brief, the 3830-69 lead (Medtronic Inc., Minneapolis, MN, USA) was sent 1-2 cm forward and downward to find the insertion point of the right side of the intraventricular septum combined with the prerotation impedance and ECG changes. Ultimately, the tip was perpendicularly straightforward and posited against the septum to the left septal side (Supplemental figure 1). If acceptable LBB area capture could not be initially achieved, the lead was repositioned at a slightly distal site. According to the LBB pacing (LBBP) capture criteria published previously [9, 10], LBB capture was confirmed using right bundle branch block (RBBB) paced morphology and one of the following signs: (1) selective LBBP (paced morphology as a typical RBBB shape with a discrete component in the intracardiac electrogram); (2) stimulus to left ventricular activation time (Sti-LVAT) shortening abruptly by >10 ms with increasing output. If only the LV septal myocardium is captured, it is called LV septal endocardium pacing (LVSP). In this retrospective cohort of LBBAP patients, approximately 60% of the included patients achieved LBBP, and approximately 40% achieved LVSP.

In the procedure of RVP, a 5076 lead (Medtronic Inc., Minneapolis, MN, USA) was positioned on the right side of the midventricular septum. Written informed consent was obtained from all participants. Clinical data, such as baseline characteristics, medical history, comorbidities, in-hospital management, and laboratory test results, were extracted according to our previous data collection process [11].

### 2.3. Clinical Outcomes and Follow-Up

The primary outcomes were composed of all-cause mortality, lead failure, and heart failure hospitalization (HFH) during the follow-up. Lead failure was defined as the reintervention for increased pacing thresholds, lead dislocation, or ventricular perforation after the initial implantation procedure [12]. HFH was defined as admission to the hospital for >24 hours with worsening symptoms and signs of heart failure and requiring one or more intravenous diuretics or intravenous inotropic medications [13, 14]. The prespecified secondary outcomes included periprocedure complication (pericardial tamponade and pneumothorax), cardiac death, recurrent unexplained syncope, ventricular pacing thresholds, and LV function. Cardiac mortality was defined as a documented arrhythmogenic death, an unexpected presumed pulseless condition with the absence of an obvious noncardiac explanation, or a death due to congestive cardiac failure or structural heart disease.

All patients were followed up via telephone interviews or clinic visits who were blind to the pacing strategy from the 25th of November 2021 to the 28th of March 2022. The patients were also invited for an echocardiographic examination at the follow-up time point. Relevant information was also collected from the clinical records of the patients who were readmitted to the hospital. For events that occurred more than once, only the index event was used for statistical analysis. Complete clinical follow-up was performed on 903 patients, while 134 were lost from the follow-up. The latest pacing parameters (including ventricular pacing threshold, pacing impedance, R wave amplitude, pacing proportion, and paced QRS duration) of pacemaker programming during the follow-up period were also collected through programming report picture uploading via questionnaires or clinic visits. A total of 217 patients completed the collection of pacing parameters.

### 2.4. Echocardiographic Examination and Analysis

Among the patients who received echocardiographic examination using an EPIQ 7 ultrasound system (Philips Medical Systems, Andover, MA, USA) during clinic visits in the follow-up, a total of 382 propensity-score matching participants (191 for each group) were assessed for echocardiographic analysis. Related parameters, such as LV end-diastolic diameter (LVEDD), LV end-diastolic volume (LVEDV), and LV ejection fraction (LVEF), were measured according to the ASE/EACVI guidelines [15]. The real-time three-dimensional echocardiography (3DE) images of the left ventricle for strain analysis were acquired in the apical 4-chamber views by EPIQ 7 with the X5-1 xMATRIX array probe. LV global systolic strain was measured with the commercially available 4D LV-Function (TomTec Imaging Systems, Munich, Germany) software (Supplemental figure 2). The specific measurements were carried out according to our previously published procedure [16]. After manually adjusting the automated contour tracing, LV global longitudinal systolic strain (GLS), global circumferential strain (GCS), global radial strain (GRS), systolic dyssynchrony index (SDI, standard deviation of the time from cardiac cycle onset to minimum systolic volume in 16 LV segments), twist, and torsion were automatically calculated. All echocardiographic parameters were calculated as the mean values of 3 consecutive cardiac cycles and 3 repeated measurements.

### 2.5. Statistical Analysis

There were no missing data for the included variables. Some variables with missing data, such as high-sensitivity cardiac troponin T and N-terminal propeptide of B-type natriuretic peptide (NT-pro BNP), were not included in our study. All analyses were performed with SPSS version 26 (IBM Corporation, Armonk, NY). All reported *p* values were 2-tailed, and *p* values < 0.05 were considered statistically significant.

Differences in outcomes were reported as odds ratios (OR) with 95% confidence intervals (CI). To address the possible imbalance in the participants' characteristics between the test groups, a propensity score approach was used. The propensity score predicting LBBAP was generated using multivariable logistic regression with LBBAP as the dependent variable and thirteen significant baseline characteristics between the test groups or traditional risk factors [age, sex, diastolic blood pressure (DBP) at admission, creatinine, diabetes mellitus, AF, hyperlipidemia, congestive heart failure, coronary artery disease, prior cerebrovascular accident, with transcatheter aortic valve implantation (TAVI), cardiac surgery, and cancer] as the independent variables. We used the propensity score as a control variable in the covariate-adjusted analysis of the outcome variables [11, 17].

For the matching cohort for echocardiographic analysis, the propensity for being in a specific treatment group was calculated using a logistic regression model with baseline covariates as follows: age, body mass index, sex, hypertension, diabetes mellitus, AF, congestive heart failure, coronary artery disease, prior cerebrovascular accident, cancer, hemoglobin, platelet, creatinine, total bilirubin, low-density lipoprotein cholesterol, and LVEF. Patients were matched in a 1 : 1 manner between the LBBAP group and the RVP group using the greedy, nearest-neighbor method without replacement with a caliper of 0.01 of the propensity score.

## 3. Results

### 3.1. Baseline Characteristics

In the total cohort, patients in the LBBAP group had a higher prevalence of AF, congestive heart failure (defined as typical heart failure symptoms and elevated NT-pro BNP), and a history of TAVI, and they also had a higher level of creatinine when compared with those patients in the RVP group. In the cohort who completed the follow-up, the baseline parameters between the two groups were similar with those in the total cohort, except that the DBP at admission was higher in patients with LBBAP. Although the median value of LVEF presented as normal, patients with LVEF < 50% were actually included in our study. The proportion of heart failure with LVEF < 50% in patients who completed follow-up and the matching cohort for echocardiographic analysis were 15.3% and 8.4%, respectively. The all-baseline characteristics were not significant between the two groups in the propensity-score-matched cohort for echocardiography. The detailed information is presented in Table 1.

### 3.2. Clinical Outcomes

Compared with the patients with the RVP regimen, the patients with the LBBAP strategy had a lower prevalence of primary outcome (5.85% vs. 10.20%, *p* = 0.019), including a lower prevalence of all-cause mortality (2.54% vs. 5.88%, *p* = 0.016). No significant difference in the secondary outcome was detected between the groups. After adjusting for the propensity scores in a logistic regression model, LBBAP was associated with a lower risk of the primary outcome (OR 0.48, 95% CI 0.28 to 0.83, *p* = 0.009). In particular, LBBAP was also independently associated with a significant reduction in mortality (OR 0.46, 95% CI 0.22 to 0.98, *p* = 0.043) and HFH (OR 0.36, 95% CI 0.14 to 0.89, *p* = 0.028) (Table 2). No significant difference in the secondary outcome was detected between the groups. After adjusting for the propensity scores in a logistic model, LBBAP showed no effect on the rates of secondary outcome except that LBBAP was associated with a lower risk of recurrent unexplained syncope (OR 0.49, 95% CI 0.26 to 0.95, *p* = 0.036) (Table 2). Additionally, in the cohort which completed the collection of pacing parameters, compared with the RVP regimen, LBBAP had a higher R wave amplitude (17.5 vs. 12.05 mV, *p* < 0.001) and ventricular pacing impedance (576.5 vs. 513.0 ohms, *p* < 0.001), while a narrower paced QRS duration (116.25 ± 16.84 vs. 149.39 ± 14.39 ms, *p* < 0.001) (Supplemental table 1).

### 3.3. Left Ventricular Systolic Synchrony

In the propensity-score matching participants assessed for echocardiographic analysis, compared with the RVP regimen, LBBAP had a higher absolute value of GLS (−19.56 ± 7.11 vs. −15.90 ± 6.67%, *p* < 0.001), GCS (−28.86 ± 6.13 vs. −26.09 ± 5.64%, *p* = 0.006), GRS, twist, and torsion. However, compared with the RVP group, we did observe a lower SDI (5.68 ± 1.92 vs. 6.50 ± 2.28%, *p* = 0.012) in the LBBP group. No significant difference in the LVEDV and LVEF was detected between the groups. The detailed information is presented in Table 3.

## 4. Discussion

Several important findings were observed in this cohort study of the safety and effectiveness performance of LBBAP in real-world clinical practices among AVB patients. Firstly, in our study, LBBAP patients had a higher prevalence of AF, congestive heart failure, and a history of TAVI, and they also had a higher level of creatinine when compared with the patients in the RVP group. Secondly, the safety endpoints of lead failure and periprocedure complications were similar between the groups. Thirdly, LBBAP was independently associated with lower risks of the primary outcome (particularly including lower risks of all-cause mortality and HFH) and recurrent unexplained syncope. Fourthly, compared with the RVP regimen, LBBAP obviously narrowed the QRS duration, and at the same time, the pacing impedance and threshold were favorable. Lastly, in the propensity-score matching cohort of echo analysis, better cardiac function was observed in LBBAP, as reflected in a higher LV strain and a lower SDI.

The phenomenon that the prevalence of AF is higher in the LBBAP group is in line with Sharma et al.'s results from the registry study [18]. Some of these differences may be attributable to the leadless devices implanted among patients with permanent AF needing single-chamber pacing, thereby decreasing the number of RVP transvenous implants included in this analysis. The higher prevalence of patients with congestive heart failure and TAVI observed in the LBBAP group may be based on operators' preference. In AVB patients with structural heart disease or congestive heart failure with no significant decrease in ejection fraction and intraventricular conduction block, operators in our center prefer LBBP in preventing the deterioration of cardiac function. Although the differences in the presentation of AF, TAVI, and congestive heart failure are linked with clinical outcomes, LBBAP is independently associated with a lower primary outcome after being adjusted with these confounders.

### 4.1. The Safety of LBBAP

Safety is the most important point to consider for a novel pacing strategy. In our study, the lead failure and periprocedure complication rate are similar between LBBAP and RVP up to 4-year follow-ups. The results suggest that LBBAP is feasible and safe among diverse AVB patients, which is consistent with Su et al.'s [8] findings. Su et al.'s study prospectively enrolled 632 consecutive pacemaker patients with attempted LBBP, 97.8% (618/632) of the patients were successful according to the strict criteria for LBB capture, and only 6 patients were observed with LBB capture threshold increasing to >3 V or loss of bundle capture (2 of the patients had a loss of conduction system capture and required a lead revision). Moreover, in Su et al.'s study, the QRS duration was significantly decreased in patients with LBB block, and the LVEF was improved in patients with QRS ≥ 120 ms. Therefore, the high success rates and low complication rates of LBBP during long-term follow-up suggest that LBBP is a feasible and reliable method of physiological pacing for patients with either bradycardia or a heart failure pacing indication. The lead failure was relatively low in our cohort, which was similar to the incidence of lead dislodgement and lead perforation in Chen et al.'s study [19, 20], while the periprocedure complication rate (included pericardial tamponade and pneumothorax) was higher than the rate of pericardial effusion reported in Chen et al.'s prospective study [19]. This may be attributed to the higher mean age and higher prevalence of structural heart disease in this real-world cohort study. In Chen et al.'s study, heart failure with LV ejection fraction < 50% and indication for cardiac resynchronization therapy were excluded.

### 4.2. The Efficacy of LBBAP

For better safety and feasibility, cardiologists also pay close attention to the effectiveness of LBBP. In our study, after having adjusted for the significant baseline characteristics and traditional risk factors, LBBAP is independently associated with lower risks of all-cause mortality, HFH, and recurrent unexplained syncope. The results are consistent with the results from the Geisinger-Rush Conduction System Pacing Registry [18]. In this registry study, LBBAP (which was considered successful if the unipolar paced QRS morphology demonstrated a Qr or qR pattern along with the recording of LBB potential, R-wave peak times in leads V_5_–V_6_ <80 ms or demonstration of transition from nonselective to selective LBB/LV capture during threshold testing) was associated with a significant reduction in the primary outcome (composed of all-cause mortality, HFH, or upgraded to biventricular pacing) compared to RVP (10.0% vs. 23.3%; HR 0.46; 95% CI 0.306-0.695; *p* < 0.001). The main drivers for the difference in the primary composite outcome between the two groups were mortality and HFH. The incidence rates of all-cause mortality and HFH are lower in the present study compared with Sharma et al.'s results [18]. This is likely due to the lower mean age (72 years vs. 75 years) and better physiological state (less comorbidities of hypertension, diabetes mellitus, AF, and coronary artery disease) of the current cohort. On the contrary, the relatively high rate of primary and secondary outcomes, compared with Chen et al.'s prospective study [19], may be attributed to the higher mean age, higher prevalence of structural heart disease, and higher prevalence of cancer in this retrospective cohort study. In our study, patients with congestive heart failure, with TAVI, with cardiac surgery, and with cancer were included. This was more in line with real-world clinical practices.

### 4.3. LBBAP Improves LV Systolic Synchrony

Additionally, LV dyssynchrony reduces myocardial efficiency because the work performed by one segment is wasted by stretching other segments [21]. Our report demonstrates that LBBAP was associated with a reduction of LV systolic dyssynchrony compared with RVP. Although the LVEF was similar between the two groups at baseline and follow-up, LBBAP tended to have a significantly higher follow-up strain, which was more sensitive to reflect cardiac function than LVEF. This may be attributed to the fact that LBBAP narrows the QRS interval, corrects the electrical abnormalities, and uncoordinates LV contractions, and thus, improves LV systolic synchrony and cardiac function. In Wang et al.'s study [22], LBBAP (75.4% of cases mapped the LBB potential) yielded a narrower paced QRS duration, a shorter QTc interval, lower QTc dispersion, and shorter *T*_peak-end_ interval than those of RVP. The better depolarization-repolarization reserve may predict a lower risk of ventricular arrhythmia and sudden cardiac death. The ability to provide a physiologic ventricular activation pattern of LBBP was also proved in Sun et al.'s study [23]. In Sun et al.'s study, ventricular systolic synchronization was implemented via 2D-STE between LBBP and RVP. The results demonstrated that both the maximum time difference and standard deviation of the 18-segment systolic time to peak systolic strain and the left and right ventricular preejection period differences were significantly shorter under LBBP than those under RVP, indicating that LBBP could provide better intra- and interventricular contraction synchronizations.

### 4.4. The Naming Debates

There are no uniform naming and criteria for this new pacing method in the initial period. Some researchers advocate naming it LBBAP [24]. With uniformity and standardization of the implant procedure and definitions, Wu et al. and Chen et al. developed the criteria and summarized the characteristics of LBBP, which was defined as pacing the proximal left bundle or its branches along with the capture of the LV septal myocardium [25, 26]. If only the LV septal myocardium is captured, it is called LVSP or deep septal pacing. Whether having the evidence of LBB capture and selective conduction pacing or not is the main difference between these two pacing regimens. LBBAP means LVSP or LBBP without clear evidence for LBB capture. For example, in the feasibility study of LBBAP performed in Vijayaraman et al.'s study and Padala et al.'s study, LBB potentials were noted, respectively, in 68% and 41% of patients [27, 28]. As LBBP might provide better interventricular synchrony by additionally capturing the rapidly conducting proximal left conduction system via implanting the pacing lead at LBB or its branches distal to the conduction lesion, it might be possible to achieve the widespread application of this form of physiological pacing. However, LBBAP is more feasible as it is not a mandatory requirement of selective LBBP because selective LBBP is sometimes difficult to achieve as LBB and its branches are not always networking distribution. Pacing the LBB area is a good alternative strategy. Although the short-term safety and effectiveness of LBBP have been proven, the long-term efficacy needs to be verified by more studies.

## 5. Limitations

This study has several limitations. Firstly, as we retrospectively enrolled patients to attempt LBBP, we were not able to precisely collect the pacing parameters during the procedure, such as LBB potential and stimulus-to-LV activation time. Thus, we could not specifically identify patients with LBBP or actually with LVSP, and we also could not perform subgroup analysis. Secondly, despite the use of the propensity score approach for controlling possible confounders, the observed results might still be subject to imbalances between the two groups due to the unmeasured confounders, such as the baseline LV strain, baseline QRS duration, and the proportion of ventricular pacing, which might also have an effect on the cardiac function. Thirdly, although the maximum follow-up time was up to 4 years and the mean follow-up time was 2.45 years, most of the follow-ups were completed retrospectively, and it was relatively difficult to verify the accurate event time. To ensure accuracy, we performed the analysis as per outcome proportions rather than time-to-event. Moreover, the relatively high rate of lost follow-up (due to no telephone response) might lead to an underestimation of the event rate. Lastly, because the baseline strain was missing, this study cannot provide delta GLS data between the baseline and follow-up, which reflected cardiac function changes more directly. However, the echocardiographic characteristics were assessed in propensity-score-matched study patients, which can reflect the protective effect of LBBP on cardiac function to a certain extent. Thus, prospective randomized studies are required to provide robust data.

## 6. Conclusions

LBBAP is a feasible novel pacing model associated with a significant reduction in the primary composite outcome of all-cause mortality, lead failure, or HFH, compared to conventional RVP. Moreover, LBBAP significantly reduces the risk of recurrent unexplained syncope and improves left ventricular systolic synchrony. More studies are necessary to investigate the long-term safety and efficacy.

## Figures and Tables

**Table 1:** Demographic and clinical characteristics of the study patients.

Parameters	Total cohort			Cohort complete follow-up			PS-matched cohort for echocardiography		
	LBBAP (*n* = 425)	RVP (*n* = 612)	*p* value	LBBAP (*n* = 393)	RVP (*n* = 510)	*p* value	LBBAP (*n* = 191)	RVP (*n* = 191)	*p* value
Mean age (yrs)	71.9 (63.90-79.40)	73.2 (62.65-79.70)	0.708	71.7 (63.73-79.47)	73.0 (61.53-79.38)	0.965	70.9 (62.5-79.4)	73.2 (60.4-79.8)	0.736
Male, *n* (%)	257 (60.47)	372 (60.78)	0.919	240 (61.07)	311 (60.98)	0.978	74 (38.74)	71 (37.17)	0.752
Body mass index (kg/m^2^)	23.50 (21.58-25.85)	23.31 (20.93-25.67)	0.100	23.48 (21.53-25.78)	23.23 (20.96-25.82)	0.169	23.52 (22.04-25.89)	23.60 (21.46-26.67)	0.936
SBP at admission (mm Hg)	139.0 ± 21.96	139.3 ± 22.69	0.842	138.9 ± 21.95	138.2 ± 22.26	0.627	141.0 ± 21.8	138.1 ± 20.3	0.177
DBP at admission (mm Hg)	74 (66-81)	72 (63-80)	0.059	74 (66-82)	72 (63-80)	0.014	74 (66-81)	73 (65-82)	0.650
Hypertension, *n* (%)	248 (58.35)	353 (57.68)	0.829	226 (57.51)	288 (56.47)	0.755	119 (62.30)	118 (61.78)	0.916
Diabetes mellitus, *n* (%)	98 (23.06)	162 (26.47)	0.213	91 (23.16)	128 (25.10)	0.499	50 (26.18)	52 (27.23)	0.817
Atrial fibrillation, *n* (%)	70 (16.47)	72 (11.76)	0.030	65 (16.54)	60 (11.76)	0.039	21 (10.99)	23 (12.04)	0.749
Hyperlipidemia, *n* (%)	29 (6.82)	35 (5.72)	0.467	28 (7.12)	31 (6.08)	0.528	15 (7.85)	12 (6.28)	0.549
CAD, *n* (%)	85 (20.0)	110 (17.97)	0.412	75 (19.08)	92 (18.04)	0.688	36 (18.85)	39 (20.42)	0.699
Prior cerebrovascular accident, *n* (%)	14 (3.29)	14 (2.29)	0.325	13 (3.31)	12 (2.35)	0.386	5 (2.62)	5 (2.62)	1.000
Congestive heart failure, *n* (%)	169 (39.76)	137 (22.39)	<0.001	152 (38.68)	105 (20.59)	<0.001	45 (23.56)	47 (24.61)	0.811
With TAVI, *n* (%)	56 (13.18)	12 (1.96)	<0.001	51 (12.98)	7 (1.37)	<0.001	/	/	
Cardiac surgery, *n* (%)	35 (8.24)	34 (5.56)	0.089	33 (9.67)	31 (6.08)	0.178	/	/	
Cancer, *n* (%)	29 (6.82)	34 (5.56)	0.401	25 (6.36)	30 (5.88)	0.765	10 (5.24)	13 (6.81)	0.519
Hemoglobin (g/L)	128 (115-142)	129 (117-142)	0.954	129 (115-142)	129 (117-143)	0.868	132 (123-146)	133 (119-145)	0.879
Platelet (×10^9^/L)	149 (114-189)	153 (122-188)	0.718	148 (114-188)	152 (121-187)	0.639	153 (116-191)	146 (122-186)	0.580
Creatinine (*μ*mol/L)	83 (69-99)	77 (64-99)	0.019	83 (70-99)	78 (64-100)	0.040	80 (68-95)	78 (66-100)	0.746
eGFR (mL/min^∗^1.73 m^2^)	75.39 (59.15-87.27)	78.66 (60.74-93.17)	0.083	75.42 (59.24-87.31)	78.79 (58.61-94.08)	0.169	79.93 (61.07-89.52)	77.45 (60.54-91.96)	0.905
Total bilirubin (*μ*mol/L)	12.9 (9.1-17.8)	11.8 (8.6-16.5)	0.051	12.8 (9.0-17.3)	11.6 (8.7-16.3)	0.087	12.4 (8.7-16.6)	11.5 (8.7-16.3)	0.504
Cholesterol (mmol/L)	3.88 (3.20-4.51)	3.81 (3.21-4.60)	0.946	3.86 (3.20-4.50)	3.80 (3.21-4.53)	0.952	4.00 ± 0.94	3.98 ± 0.91	0.807
LDL-C (mmol/L)	2.14 (1.60-2.74)	2.14 (1.62-2.68)	0.984	2.14 (1.61-2.73)	2.14 (1.61-2.67)	0.930	2.22 ± 0.78	2.26 ± 0.73	0.564
LVEDD (mm)	49 (46-53)	48 (45-52)	0.103	49 (46-53)	48 (45-52)	0.256	49 (46-51)	49 (46-52)	0.412
LVEF, %	65.0 (59.0-70.0)	65.0 (60.0-70.0)	0.433	65.0 (59.0-70.0)	65.5 (60.0-70.0)	0.431	66.0 (61.0-70.0)	65.0 (60.0-70.0)	0.589

CAD: coronary artery disease; DBP: diastolic blood pressure; eGFR: estimated glomerular filtration rate; LDL-C: low-density lipoprotein cholesterol; LVEDD: left ventricular end-diastolic diameter; LVEF: left ventricular ejection fraction; LBBP: left bundle-branch pacing; PS: propensity score; RVP: right ventricular pacing; SBP: systolic blood pressure; TAVI: transcatheter aortic valve implantation.

**Table 2:** The association between LBBAP and the occurrence of clinical outcomes.

Variables	LBBAP (*n* = 393)	RVP (*n* = 510)	*p* value	Adjusted odds ratio (95% CI)^∗^	*p* value
Primary outcome: all-cause mortality, lead failure, and HFH	23 (5.85)	52 (10.20)	0.019	0.48 (0.28-0.83)	0.009
All-cause mortality	10 (2.54)	30 (5.88)	0.016	0.46 (0.22-0.98)	0.043
Lead failure	6 (1.53)	5 (0.98)	0.663	1.75 (0.46-6.75)	0.414
HFH	7 (1.78)	17 (3.33)	0.151	0.36 (0.14-0.89)	0.028
Secondary outcome: periprocedure complication, cardiac death, and recurrent unexplained syncope	36 (9.16)	55 (10.78)	0.422	0.63 (0.39-1.00)	0.051
Periprocedure complication	19 (4.83)	20 (3.92)	0.503	1.08 (0.54-2.14)	0.837
Cardiac death	3 (0.76)	7 (1.37)	0.585	0.45 (0.09-2.28)	0.334
Recurrent unexplained syncope	16 (4.07)	29 (5.69)	0.269	0.49 (0.26-0.95)	0.036

LBBAP: left bundle-branch area pacing; RVP: right ventricular pacing; HFH: heart failure hospitalization. ^∗^Adjusted for the propensity score in regression models.

**Table 3:** Echocardiographic characteristics of the PS-matched study patients.

Variables	LBBAP (*n* = 191)	RVP (*n* = 191)	*p* value
LVEDV (mL)	97.62 ± 52.29	105.73 ± 47.48	0.338
LVEF, %	61.90 (56.40-65.65)	60.00 (50.10-65.52)	0.074
GLS, %	−19.56 ± 7.11	−15.90 ± 6.67	<0.001
GCS, %	−28.86 ± 6.13	−26.09 ± 5.64	0.006
GRS, %	41.97 (32.32-46.15)	34.8 (28.40-38.99)	<0.001
Twist (°)	11.10 (7.30-16.75)	9.25 (5.20-12.43)	0.045
Torsion (°/cm)	1.45 (0.82-2.10)	1.06 (0.60-1.65)	0.016
SDI, %	5.68 ± 1.92	6.50 ± 2.28	0.012

LBBAP: left bundle-branch area pacing; RVP: right ventricular pacing; LVEDV: left ventricular end-diastolic volume; LVEF: left ventricular ejection fraction; GLS: global longitudinal strain; GCS: global circumferential strain; GRS: global radial strain; PS: propensity score; SDI: systolic dyssynchrony index.

## Data Availability

The data underlying the findings of the paper are freely available on request through the authors themselves. Xingbin Liu (Department of Cardiology, West China Hospital of Sichuan University, 37 Guoxue Xiang, Chengdu, Sichuan 610041, China. Email: liuxingbin@163.com) should be contacted to request the data.

## Supplementary Materials

Supplementary 1 Supplemental figure 1: pacing lead location and ECG characteristics. Supplemental figure 2: representative images of the left ventricular global longitudinal systolic strain analysis.

Supplementary 2 Supplemental table 1: pacing characteristics of the study patients completed the collection of pacing parameters.
