# Supplementary material for: Left Bundle Branch Area Pacing versus Right Ventricular Pacing in Patients with Atrioventricular Block: An Observational Cohort Study
Source: Cardiovasc Ther. 2023 Aug 21;2023:6659048. doi: 10.1155/2023/6659048 (PMC10462439; doi:10.1155/2023/6659048)
Supplement: Supplementary 1 — Supplemental figure 1: pacing lead location and ECG characteristics. Supplemental figure 2: representative images of the left ventricular global longitudinal systolic strain analysis. [file 6659048.f1.pdf]

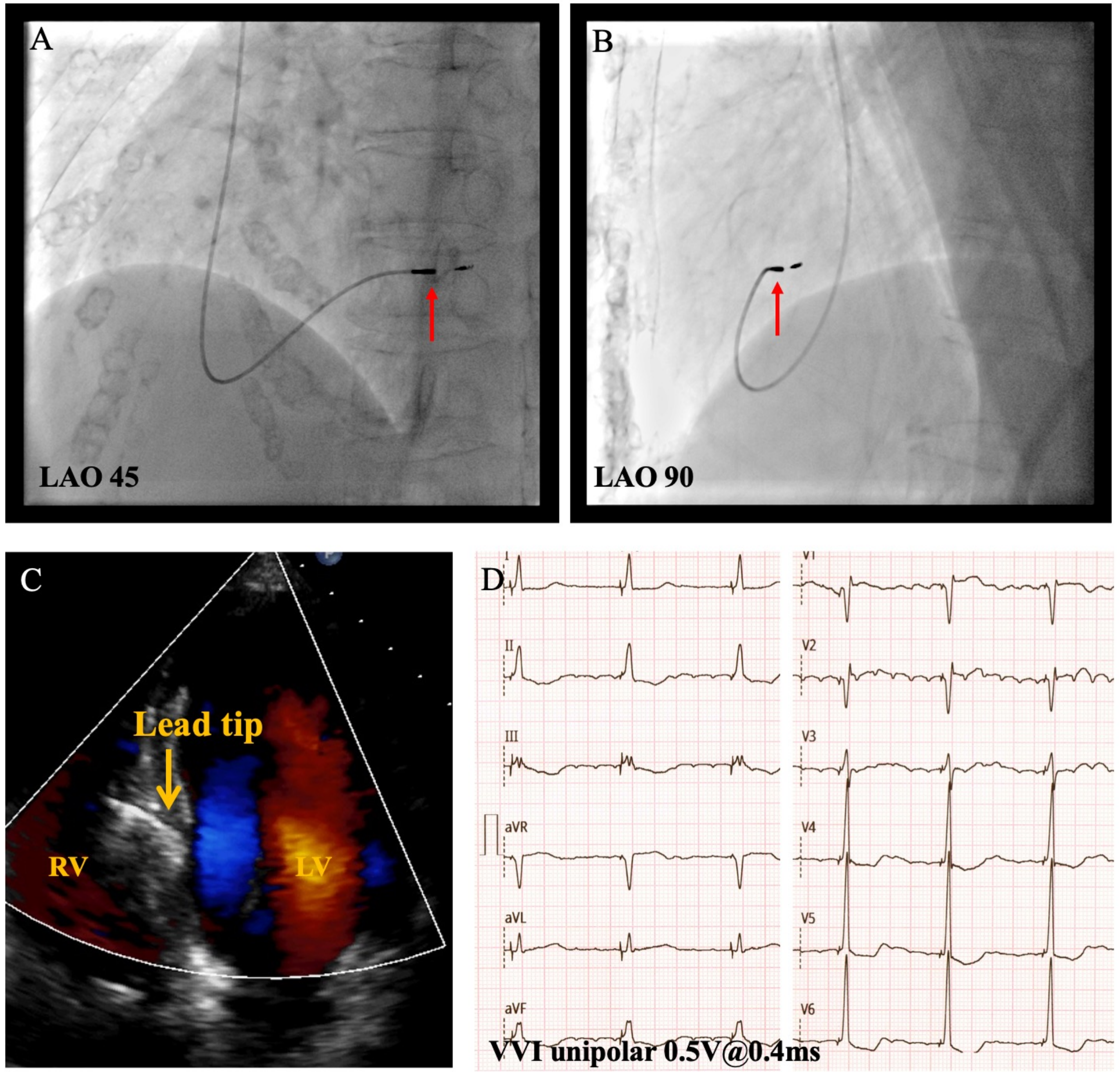

Supplemental figure 1. **Pacing lead location and ECG characteristics.** Upper Panel: LAO 45° (A) and LAO 90° (B) fluoroscopy, and post-implant echocardiography (C) confirmed the location of the LBBAP lead (red and orange arrow) on the left side of the basal septum. The ECG characteristics post-LBBAP (D). LAO: left anterior oblique; LV: left ventricle; RV: right ventricle.

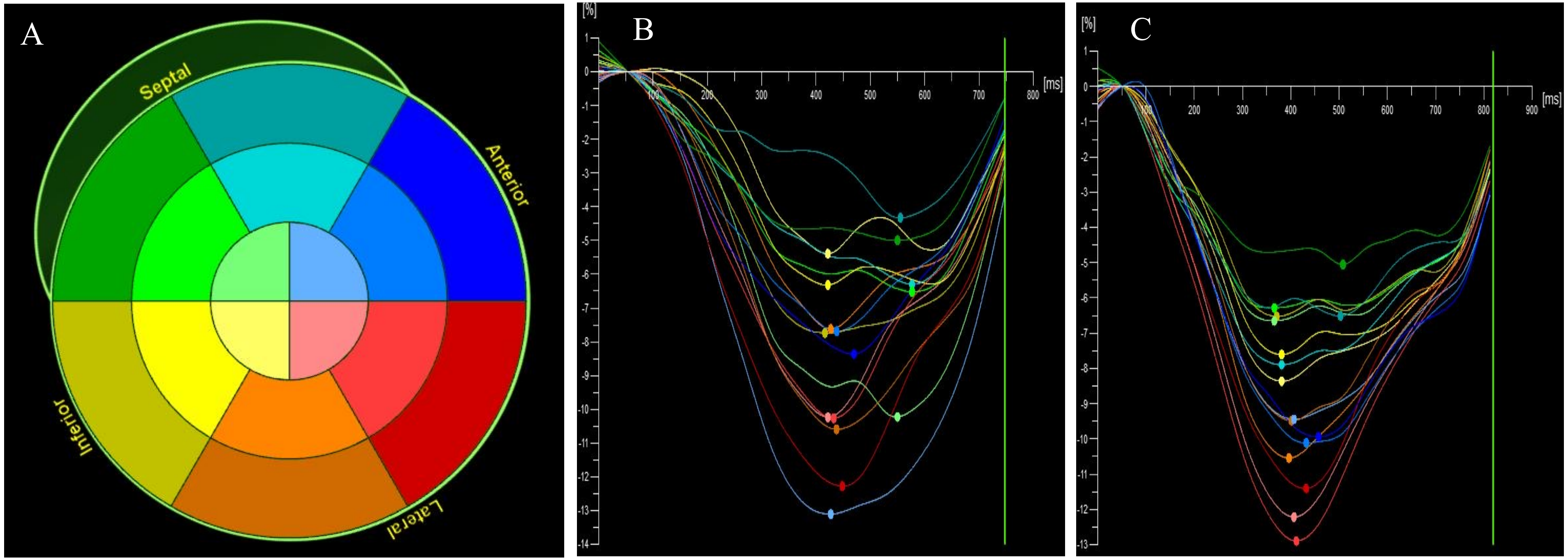

Supplemental figure 2. Representative bull's-eye map of 16 LV segments (A) and representative images of the left ventricular (LV) global longitudinal systolic strain analysis in a patient with right ventricular pacing (B) and left bundle branch pacing (C). The analyses were performed using commercially available 4D LV-Function software (TomTec Imaging Systems, Munich, Germany).
