# Supplementary material for: Left Bundle Branch Area Pacing versus Right Ventricular Pacing in Patients with Atrioventricular Block: An Observational Cohort Study
Source: Cardiovasc Ther. 2023 Aug 21;2023:6659048. doi: 10.1155/2023/6659048 (PMC10462439; doi:10.1155/2023/6659048)
Supplement: Supplementary 2 — Supplemental table 1: pacing characteristics of the study patients completed the collection of pacing parameters. [file 6659048.f2.docx]

Supplemental table 1. Pacing characteristics of the study patients completed the collection of pacing parameters.

| **Variables** | **LBBAP (n = 96)** | **RVP (n = 121)** | **P value** |
| --- | --- | --- | --- |
| Ventricular pacing threshold, V at 0.4 ms | 1.00 (0.75-1.00) | 0.84 (0.75-1.00) | 0.062 |
| R wave amplitude, mV | 17.5 (15.68-20.00) | 12.05 (8.00-20.00) | <0.001 |
| Ventricular pacing impedance, ohms | 576.5 (526.0-646.0) | 513.0 (473.5-571.5) | <0.001 |
| Patients with > 40%  ventricular pacing, n (%) | 80 (83.3) | 103 (85.1) | 0.719 |
| Paced QRS duration, ms | 116.25 ± 16.84 | 149.39 ± 14.39 | <0.001 |

LBBAP: left bundle-branch area pacing; RVP: right ventricular pacing.
